# Room Temperature Strengthening and High-Temperature Superplasticity of Mg-Li-Al-Sr-Y Alloy Fabricated by Asymmetric Rolling and Friction Stir Processing

**DOI:** 10.3390/ma16062345

**Published:** 2023-03-15

**Authors:** Furong Cao, Chao Xiang, Shuting Kong, Nanpan Guo, Huihui Shang

**Affiliations:** 1 School of Materials Science and Engineering, Northeastern University, Shenyang 110819, China; 2 State Key Laboratory of Rolling and Automation, Northeastern University, Shenyang 110819, China; 3 AVIC Xi’an Aircraft Industry Group Company Ltd., Xi’an 710089, China

**Keywords:** Mg-Li-Al-Sr-Y alloy, asymmetric rolling, friction stir processing, microstructure, superplasticity, diffusion, deformation mechanism

## Abstract

Magnesium-lithium alloy is the lightest alloy to date. To explore its room temperature strength and high-temperature ductility, a plate of a new fine-grained Mg-9.13Li-3.74Al-0.31Sr-0.11Y alloy was fabricated by asymmetric rolling, and the rolled plate was subjected to friction stir processing (FSP). The microstructure and mechanical properties at room and elevated temperatures were investigated by optical microscopy, X-ray diffraction (XRD), energy dispersive spectroscopy (EDS), and tensile tester. Grain refinement with an average grain size in the α-Mg phase of 1.65 μm and an average grain size in the β-Li phase of 4.24 μm was achieved in the water-cooled FSP alloy. For room temperature behavior, the ultimate tensile strength of 208 ± 4 MPa, yield strength of 193 ± 2 MPa, and elongation of 48.2% were obtained in the water-cooled FSP alloy. XRD and EDS analyses revealed that the present alloy consists of α-Mg and β-Li phases, Al_2_Y, Al_4_Sr, MgLi_2_Al, and AlLi intermetallic compounds. For high-temperature behavior, the maximum superplasticity or ductility of 416% was demonstrated in this fine-grained alloy with an average grain size of 10 μm at 573 K and 1.67 × 10^−3^ s^−1^. A power-law constitutive equation was established. The stress exponent was 2.29 (≈2) (strain rate sensitivity 0.44), and the deformation activation energy was 162.02 kJ/mol. This evidence confirmed that the dominant deformation mechanism at elevated temperatures is grain boundary and interphase boundary sliding controlled by lattice diffusion.

## 1. Introduction

Magnesium-lithium (Mg-Li) alloys have found certain applications in the automobile industry, 3C (Computer, Communication, and Consumer Electronic) electronics, weapons, and spaceflight industries owing to their extremely low density, excellent specific stiffness, good specific strength, good electromagnetic shielding properties, and damping properties. Due to the demand of aviation and spaceflight industries, studies on the room temperature microstructure, mechanical properties, and strengthening have been reported in some Mg-Li alloys [1,2,3,4]. Meanwhile, investigations into the superplasticity and hot deformation of Mg-Li alloys attract extensive attention because aerospace environment sustains the temperature difference up to 423 K, and high-temperature performance is urgently required [5,6,7,8,9,10,11,12,13,14,15]. Table 1 summarizes the grain refinement and superplasticity of Mg-Li-based alloys, indicating that the superplasticity of Mg-Li alloys captured the attention of researchers. Hence, a novel dual-phase multicomponent Mg-9Li-4Al-0.5Sr-0.5Y alloy was designed and fabricated, and its room and high-temperature deformation behaviors were studied.

Severe plastic deformation has been extensively studied over past decades [16,17]. In Mg-Li alloys, Kong et al. [18] used constrained groove pressing to achieve grain refinement in an Mg-7.91Li-1.34Al alloy. Rahmatabadi et al. [19] utilized accumulative roll bonding to produce an AA1050/MgLZ91 laminate. Specifically, friction stir processing (FSP), as one of the severe plastic deformation approaches, is a further development of friction stir welding and has captured widespread attention. FSP utilizes a stirring head to drive the stirring pin into the metallic plate and cause intense dynamic recrystallization and intense plastic deformation [20,21]. As a result, significant grain refinement is achieved, and the mechanical properties are improved greatly. According to our survey, reports [22,23,24,25,26,27,28] about the superplasticity of magnesium alloys were documented because of the grain refinement by FSP. Table 2 summarizes the grain refinement and superplasticity of magnesium alloys processed by FSP, indicative of researcher interest. In particular, two reports about the superplastic flow behavior of Mg-Li alloys processed by cross-flat rolling + FSP and extrusion + FSP [29,30] are available. Both reports paid more attention to the superplastic deformation behavior and microstructural evolution, and the elucidation of the underlying mechanism did not receive enough attention. Thus, the superplastic behavior and microstructures and the elucidation of the deformation mechanism in the Mg-9Li-4Al-0.5Sr-0.5Y alloy processed by asymmetric rolling + FSP remain unknown. Superplasticity reflects the capability of materials to exhibit exceptional ductility or elongation [31]. Complex-shaped components can be formed under a small load using superplastic forming [32,33]. Thus, it is essential to study the superplastic behavior and expound the deformation mechanism in the Mg-9Li-4Al-0.5Sr-0.5Y alloy subjected to asymmetric rolling + FSP.

In this work, our aims include several aspects. Firstly, a novel Mg-9Li-4Al-0.5Sr-0.5Y alloy is fabricated by asymmetric rolling + FSP. Secondly, the microstructures and mechanical properties at room and elevated temperatures are studied. Thirdly, the room temperature strengthening mechanism and high-temperature superplasticity mechanism are expounded.

## 2. Experimental Procedures

### 2.1. Alloy Preparation

**Figure 1:**
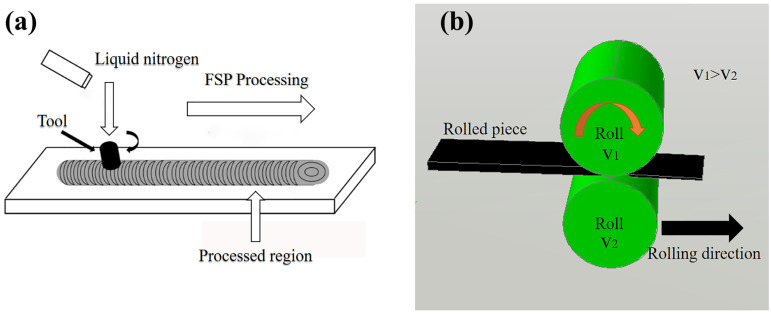
Schematic diagram of (**a**) FSP and (**b**) asymmetric rolling.

The melting and casting of the Mg-Li-Al-Sr-Y alloy were performed using Jackson’s approach. The flux (LiCl + LiF) coverage and argon atmosphere were adopted to prevent lithium vaporization. The analyzed chemical composition was Mg-9.13Li-3.74Al-0.31Sr-0.11Y in wt.% (hereinafter denoted as LASY9400 alloy). The ingot was homogenized at 523 K for 20 h. After milling the ingot surface, the milled ingot was asymmetrically rolled at 523 K to 6 mm thick with a reduction of 75%. 75% thickness reduction was applied in six passes. Then the rolled plates were subjected to friction stir processing. The schematic diagram of the FSP is shown in Figure 1a and elsewhere [34], and the schematic diagram of asymmetric rolling is shown in Figure 1b. The conical stirring head was used, with a pin diameter of 2.7 mm, root diameter of 4.7 mm, and shoulder diameter of 16 mm. The FSP parameters were that the rotating speed was 600 rpm, the transverse speed was 100 mm/min, and the plunging depth was 0.2 mm. During FSP, the stirring pin was slowly inserted into the rolled plate until the plunging depth. Then the forward and transverse moving were carried out to induce intense plastic deformation.

### 2.2. Tensile Tests

Dog bone samples were spark discharge machined from the FSPed plate perpendicular to the forward direction of FSP. The sample dimensions were 3 mm (gauge length) × 2 mm (gauge width) × 2 mm (gauge thickness). The tensile test velocity at room temperature was 0.3 mm/min. The number of testing samples was three. After being held at the designated temperature for 15 min, high-temperature tensile tests were performed for water-FSP samples in a SANS-CMT5105 universal tensile tester in the temperature range of 473–623 K over the strain rates of 1.67 × 10^−2^–1.67 × 10^−4^ s^−1^**.** After tension, the tensile samples were quenched into water to preserve the high-temperature microstructures. 

### 2.3. Microstructural Characterization

The samples for optical microstructure observation were ground, polished, and etched as per the conventional metallographic method. The etching solution was 4% HCl and 96% ethanol. The optical examination was carried out on an Olympus DSX500 optical microscope. Image-Pro-Plus (IPP) software was used to measure the grain size and phase volume fraction. 

The samples for X-ray diffraction (XRD) detection were ground and polished to obtain a flat surface. The scanning angle was 10–90°. The scanning velocity was 6°/min. The voltage was 40 kV. The current was 40 mA. The XRD test was carried out on the X’Pert Pro X-ray diffractometer. Jade 6.5 software was used to analyze the phase composition.

The scanning electron microscopy (SEM) samples were taken from the XRD samples. SEM observations and energy dispersive spectroscopy (EDS) analysis were conducted on an SSX-550 scanning electron microscope.

## 3. Results

### 3.1. Microstructures and Mechanical Properties of This Alloy at Room Temperature

**Figure 2:**
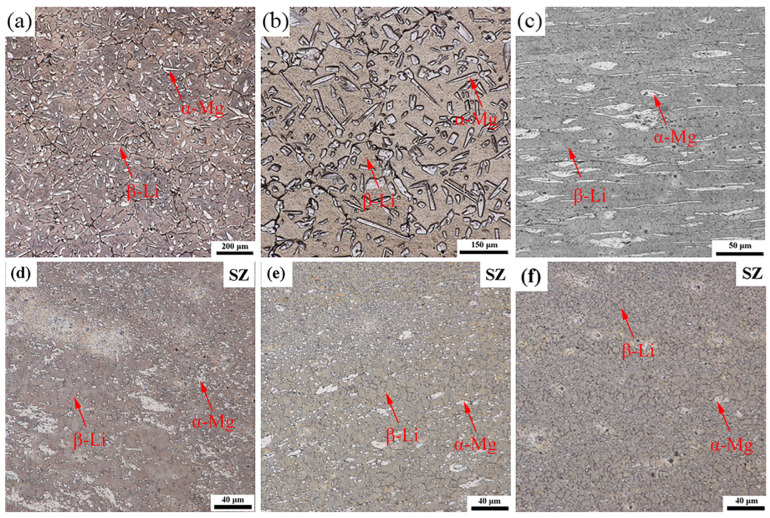
Optical microstructures of LASY9400 alloy in different states: (**a**) as-cast state, (**b**) homogenized at 523 K for 20 h, (**c**) as-rolled state, horizontal rolling direction, (**d**) friction stir processing cooled in air, stir zone (SZ), (**e**) friction stir processing cooled in liquid nitrogen, SZ, and (**f**) friction stir processing cooled by water, SZ.

Figure 2 presents the optical microstructures of LASY9400 alloy in different states. As shown in Figure 2a, the as-cast microstructure was composed of an acicular white α-Mg solid solution phase and a yellow and gray blocky solid solution β-Li phase. The phase proportion of the α-Mg phase to the β-Li phase was measured as 26.3:73.7, indicating that the β-Li phase is the matrix phase. Some big black particles are dispersed in the β-Li phase matrix. As shown in Figure 2b, the as-homogenized microstructure eliminates the element segregation, and the grain boundary becomes rounded and clear. Also, some big black particles are still dispersed in the β-Li phase matrix without dissolution in the matrix. As shown in Figure 2c, under the applied rolling force, the grains are flattened, fragmented, and elongated along a horizontal rolling direction. The average grain size, usually the average bandwidth, is 2 μm. This indicates that fine grains are obtained due to the shear stress refinement exerted by the asymmetric rolling. As shown in Figure 2d, due to FSP cooling in air, grain refinement occurs in the stir zone (SZ, marked in the image). This grain refinement is evidence of dynamic recrystallization and plastic deformation compared to Figure 1c. The grain size was measured by Image-Pro-Plus (IPP) software for Figure 1d. The average grain size in the α-Mg phase was 1.78 μm, and the average grain size in the β-Li phase was 5.04 μm. As shown in Figure 2e, after FSP cooling by liquid nitrogen, the average grain size of the α-Mg phase was 1.45 μm, and the average grain size of the β-Li phase was 4.08 μm. Compared with Figure 2d, nitrogen-FSP led to more grain refinement than air-FSP. As shown in Figure 2f, after FSP cooling by water, the average grain size in the α-Mg phase was 1.65 μm, and the average grain size in the β-Li phase was 4.24 μm. This reflects the grain refinement of the α-Mg phase due to FSP processing and grain coarsening of the β-Li phase due to deformation heat generated by FSP. This also indicates that grain refinement does not vary much for the present dual-phase alloy after it is treated by three cooling modes. Even so, the FSP microstructures are fine-grained microstructures that favor the improvement of mechanical properties at room temperature and provide a prerequisite for the occurrence of superplasticity at elevated temperatures. A further discussion will be given in Section 4.1 and Section 4.2.

**Figure 3:**
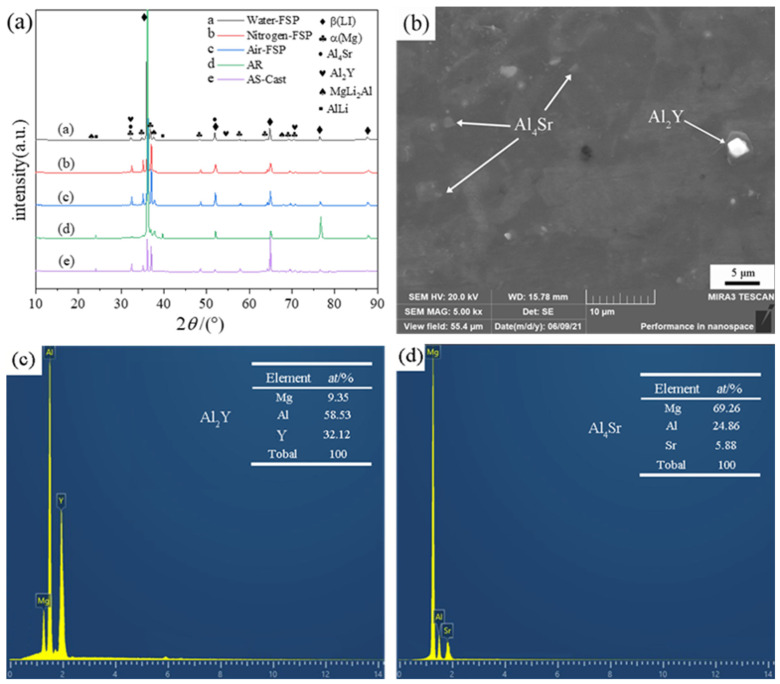
Phase composition analysis of LASY9400 alloy: (**a**) XRD, (**b**) SEM, (**c**) EDS of Al_2_Y, and (**d**) EDS of Al_4_Sr.

Figure 3 presents the results of the phase composition analysis of the LASY9400 alloy. Water-FSP, Nitrogen-FSP, and Air-FSP mean that the FSP treatments are conducted under water spray, nitrogen-cooling, and air, respectively. As shown in Figure 3a, analysis of XRD reveals that the present alloy consists of α-Mg and β-Li phases and Al_2_Y, Al_4_Sr, MgLi_2_Al, and AlLi intermetallic compounds. The α-Mg and β-Li phases achieve solid solution strengthening during processing. Al_2_Y and Al_4_Sr compounds possess high melting points and can realize the second phase strengthening during processing. MgLi_2_Al is a metastable strengthening compound during thermal processing and can be transformed into a soft AlLi compound phase at room temperature. As shown in Figure 3b, the SEM image reveals the morphologies of Al_2_Y and Al_4_Sr compounds. As shown in Figure 3c,d, EDS results demonstrate the existence of Al_2_Y and Al_4_Sr compounds. 

**Figure 4:**
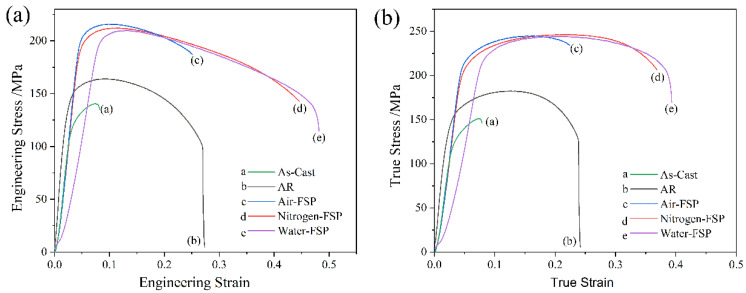
Stress-strain curves in LASY9400 alloy under different processing conditions: (**a**) engineering stress-engineering strain curves, and (**b**) true stress-true strain curves.

Figure 4 presents the stress-strain curves in LASY9400 alloy under different processing conditions. Table 3 lists the room-temperature mechanical property data. As shown in Figure 4a, the as-cast alloy has the lowest ultimate tensile strength (UTS) of 141 ± 2 MPa, yield strength (YS) of 133 ± 4 MPa, and elongation (EL) of about 8.2%. After asymmetric rolling, the UTS, YS, and EL of 164 ± 3 MPa, 113 ± 3 MPa, and 27.3% are obtained. After air-FSP, the UTS is 215 ± 3 MPa, the YS is 207 ± 2 MPa, and the EL is 25.4%. Compared with the mechanical properties of asymmetric rolling, the UTS of the air-FSP alloy increases by 30%, and the EL varies little, which indicates FSP enhances the mechanical properties of the present alloy. The UTS and YS data of the nitrogen-FSP alloy and water-FSP alloy do not vary much and are 212 ± 2 MPa and 202 ± 1 MPa and 208 ± 4 MPa and 193 ± 2 MPa, respectively. Still, the EL data of the nitrogen-FSP alloy and water-FSP alloy are much higher and are 44.6% and 48.2%, respectively. During severe plastic deformation, grain refinement, dislocation strengthening, second phase strengthening, and solid solution strengthening occur in the alloy. These strengthening routes contribute to improving its mechanical properties. As shown in Figure 4b, with the increase in true strain, the true stress increases for asymmetric rolling and FSP alloys until the peak stress. However, after the peak stress, true stress decreases, which indicates strain softening relevant to the β-Li matrix, and usually occurs in Mg-Li alloy with Li content of more than 9 wt.%.

### 3.2. Microstructures and Mechanical Properties of This Alloy at Elevated Temperatures

**Figure 5:**
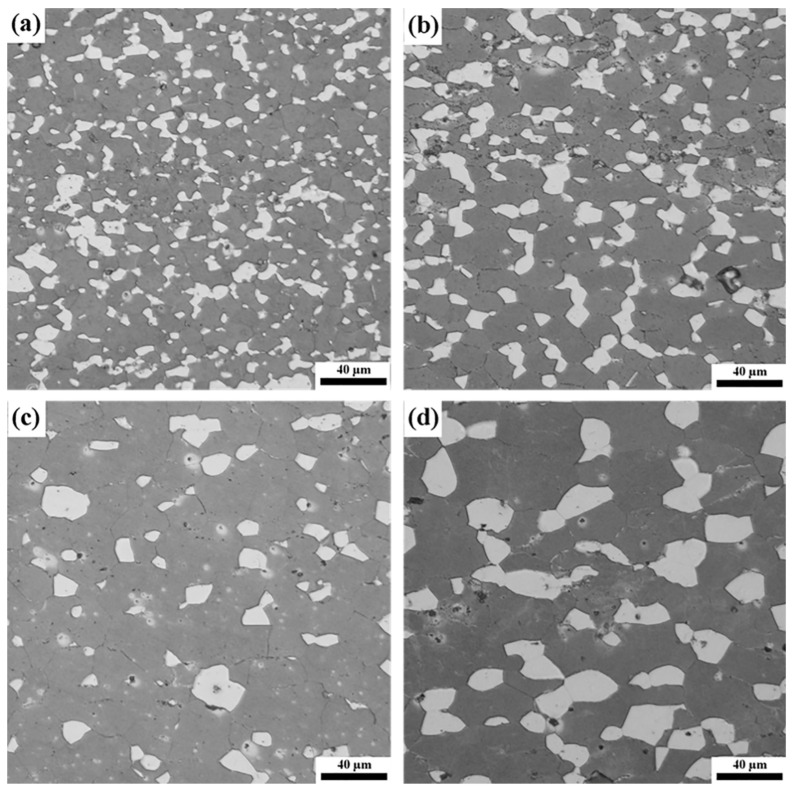
Microstructures of LASY9400 alloy in the gauge section at the tensile deformation temperature of 573 K and different strain rates: (**a**) 1.67 × 10^−2^ s^−1^, δ = 317%, *d* = 6.81 μm; (**b**) 1.67 × 10^−3^ s^−1^, δ = 416%, *d* = 10 μm; (**c**) 5.0 × 10^−4^ s^−1^, δ = 257%, *d* = 15.62 μm; (**d**) 1.67 × 10^−4^ s^−1^, δ = 335%, *d* = 17.35 μm. δ indicates the elongation, while *d* indicates the grain size.

Figure 5 presents the microstructures of LASY9400 alloy in the gauge section at the tensile deformation temperature of 573 K and different strain rates. With the decrease in strain rate, thermal deformation time prolongs, atomic diffusion accelerates, and grain coarsening occurs, with an average grain size ranging from 6.81 to 17.35 μm. In particular, the maximum superplasticity or ductility of 416% is demonstrated in this fine-grained alloy with an average grain size of 10 μm at 573 K and 1.67 × 10^−3^ s^−1^, as shown in Figure 5b. In the meantime, isolated black cavities are visible in the microstructures following superplastic tension.

**Figure 6:**
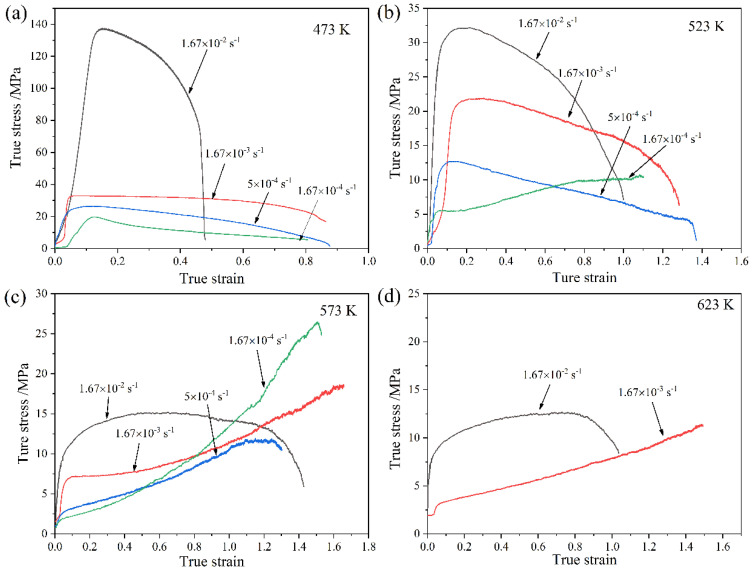
True stress versus true strain curves of LASY9400 alloy with different strain rates at the given temperature: (**a**) 473 K; (**b**) 523 K; (**c**) 573 K; (**d**) 623 K.

Figure 6 presents the true stress versus the true strain curves of the LASY9400 alloy with different strain rates at the given temperature. At the given temperature, flow stress, in most cases, decreases with decreasing the strain rate because a lower strain rate enhances the deformation time, thermal activation accelerates, and hence the flow stress decreases. At the given strain rate, the flow stress decreases with increasing the temperature because higher tensile temperature enhances the deformation energy, thermal activation accelerates, and hence the flow stress decreases. As shown in Figure 6a,b, the flow curves after the peak stress exhibit typical strain-softening curves and indicate the occurrence of dynamic recrystallization. However, the flow stress curve at 523 K and 1.67 × 10^−4^ s^−1^ exhibits strain hardening. As shown in Figure 6c,d, the flow curves exhibit strain-hardening curves and indicate the occurrence of deformation-induced grain coarsening, which is corroborated by Figure 5. However, the flow stress curve at 573 K and 1.67 × 10^−2^ s^−1^ exhibits strain softening after the peak stress. This issue will be discussed in Section 4.2.

### 3.3. Power-Law Constitutive Equation at Elevated Temperatures

To deepen understanding of the deformation mechanism at elevated temperatures, a power-law constitutive equation is established and is suitable for application in superplastic forming process control. Power-law constitutive equation at elevated temperature is generally given by [35,36]:

(1) $$\dot{\varepsilon }=\frac{AD_{0}Gb}{kT}{\left(\frac{b}{d}\right)}^{p}{\left(\frac{\sigma -\sigma_{0}}{G}\right)}^{n}\exp\left(-\frac{Q}{RT}\right)$$

where $\dot{\varepsilon }$ is the steady-state deformation rate, *A* is a dimensionless constant, *G* is the shear modulus, a function of temperature, *b* is the magnitude of Burgers vector of dislocation, *b* (Mg) = 3.21 × 10^−10^ m [37], *k* is Boltzmann’s constant, *k* = 1.38 × 10^−23^ J/K, *T* is the absolute temperature, *d* is the grain size, *p* is the grain size exponent, *σ* is the applied stress, $\sigma_{0}$ is the threshold stress, *n* is the stress exponent (1/*m*, *m*-strain rate sensitivity index), *D*_0_ is the frequency factor for diffusion, *Q* is the deformation activation energy, and *R* is the universal gas constant. Here, the *n*-value, *p*-Value, and *Q*-value will be determined. The shear modulus of *G* for pure Mg is given by [38]:

(2) $$G\left(\mathrm{MPa}\right)=1.66\times 10^{4}\left[1-0.49\left(\frac{T-300}{924}\right)\right]$$

#### 3.3.1. Threshold Stress $\sigma_{0}$ and Stress Exponent *n*

**Figure 7:**
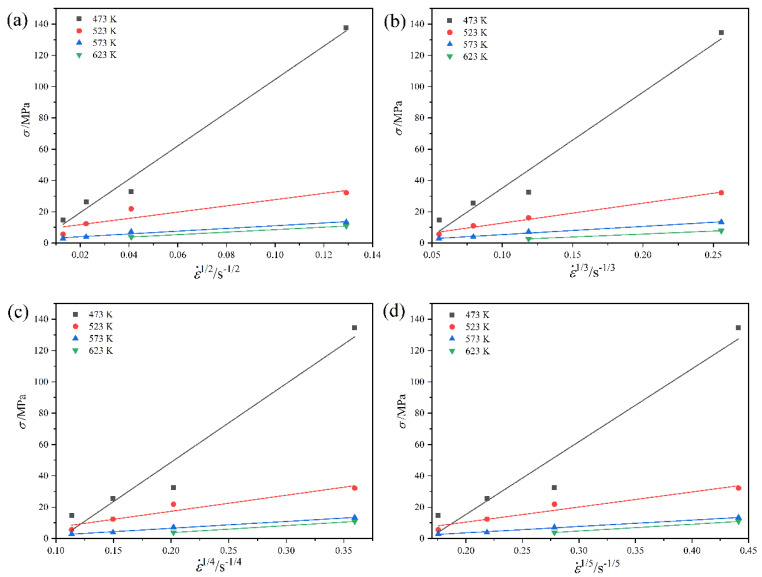
Linear fitting of σ versus $\dot{\varepsilon }$^1/*n*^ for the LASY9400 alloy: (**a**) *n* = 2; (**b**) *n* = 3; (**c**) *n* = 4; (**d**) *n* = 5.

Threshold stress $\sigma_{0}$ is the onset stress to initiate plastic flow in the alloy containing the second phases. Figure 7 shows the linear fitting of σ–$\dot{\varepsilon }$^1/*n*^ relation in this alloy to determine the threshold stress and stress exponent. At the true strain of 0.2, the values of threshold stress $\sigma_{0}$ are determined at zero strain rate using linear fitting of σ–$\dot{\varepsilon }$^1/*n*^ relation. The stress exponents are taken as 2, 3, 4, and 5. When *n* ≥ 3, the threshold stresses become negative. Hence, *n* = 3, 4, and 5 are excluded. The determination coefficient, *R*^2^, of 0.9888 with high correlation accuracy when *n* = 2. Thus, the true stress exponent is determined to be 2. 

#### 3.3.2. Deformation Activation Energy *Q* and Grain Size Exponent *p*

According to Equation (1), the deformation activation energy is given by:

(3) $$Q=R{\frac{\partial \left[\ln\left(\sigma^{n}G^{1-n}T^{-1}d^{-p}\right)\right]}{\partial \left(T^{-1}\right)}|}_{\dot{\varepsilon }}$$

The deformation mechanism in the present alloy is grain boundary sliding because the maximum elongation of 416% at 573 K and 1.67 × 10^−3^ s^−1^ is more than 400% [39]. Thus, the *p*-value should be determined to check whether *p* = 2 for lattice diffusion or *p* = 3 for grain boundary diffusion. The effective diffusion coefficient is given by [40]:

(4) $$D_{eff}=D_{l}+xf_{gb}D_{gb}$$

where *x* = 1 × 10^−2^ for superplasticity, *f_gb_* = πw/d, *w* is the grain boundary width, *w* = 2*b*. As per our previous model [41], the lattice diffusion coefficient, $D_{l}$, and grain boundary diffusion coefficient, $D_{gb}$, are given by:

(5) $$D_{l}=0.779\times 10^{-4}exp\left(-1.55\times 10^{4}/T\right)+0.221\times 2.5\times 10^{-4}exp\left(-1.24\times 10^{4}/T\right)$$

(6) $$D_{gb}=0.779\times 10^{-4}exp\left(-8.26\times 10^{3}/T\right)+0.221\times 10^{-4}exp\left(-8.11\times 10^{3}/T\right)$$

The grain sizes are shown in Table 4. The atomic diffusion mechanism is judged by the following relation:

(7) $$\phi =\frac{xf_{gb}D_{gb}}{D_{l}}$$

**Figure 8:**
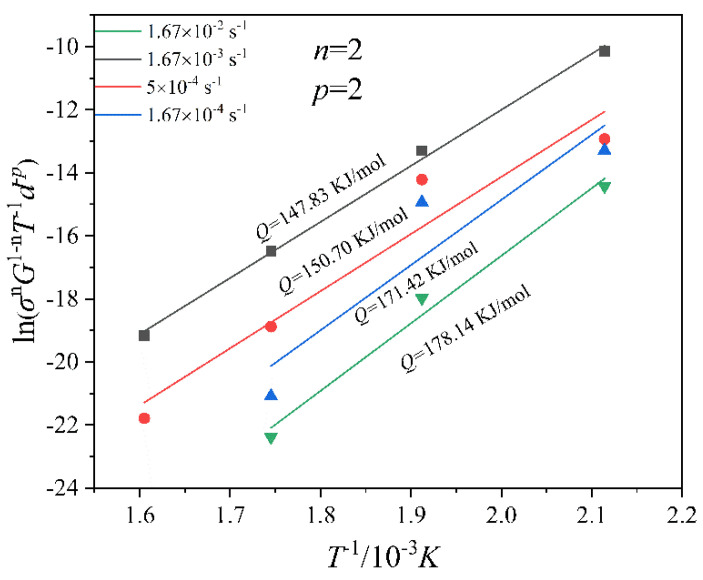
Fitting curves of $ln\left(\sigma^{n}G^{1-n}T^{-1}d^{-p}\right)-1/T$ at different strain rates.

When ϕ < 1, lattice diffusion dominates, whereas ϕ > 1, grain boundary diffusion dominates. After calculation, ϕ is in the range of 0.001~0.06 in this alloy in the temperature range of 473–623 K. Thus, the dominant diffusion mechanism is lattice diffusion. Hence, *p* = 2 as per reference [42]. Substitution of the aforementioned data into Equation (3) gives *Q =* 147.83~178.14 kJ/mol, as shown in Figure 8. The average experimental activation energy is *Q* = 162.02 kJ/mol. 

The diffusion coefficient is given by $D=D_{0}exp\left(\frac{-Q}{RT}\right)$, *D*_0_ = 1.0 × 10^−4^ m^2^·s^−1^ [43]. Substitution of Equations (5) and (6) into the above relation results in Table 5. Table 5 shows that, compared with the average experimental activation energy of 162.02 kJ/mol, the theoretical activation energy is 105.42–106.12 kJ/mol (≈105.77 kJ/mol), indicating that the diffusion mechanism is lattice diffusion.

#### 3.3.3. Normalized Curve

**Figure 9:**
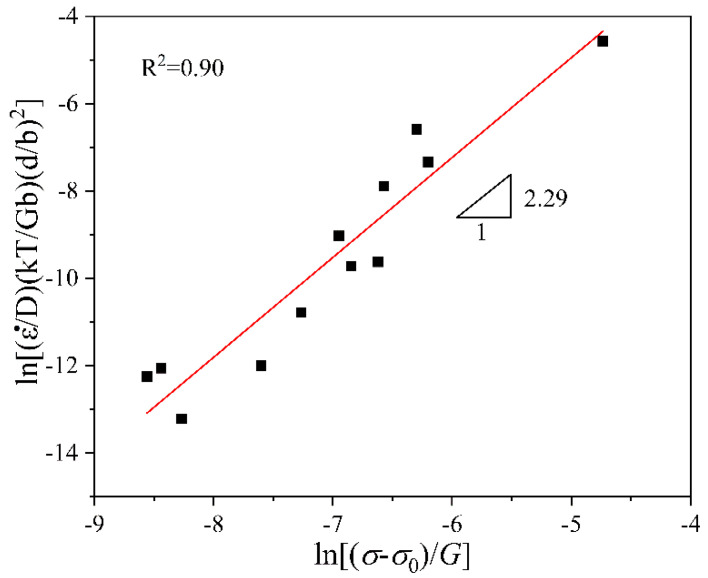
Fitting curve of $\ln\left[\left(\dot{\varepsilon }/D\right)\left(kT/Gb\right){\left(d/b\right)}^{2}\right]-ln\left[\left(\sigma -\sigma_{0}\right)/G\right]$. The square points are normalized experimental data, and the red line is the fitting line.

Figure 9 shows the normalized curve of $\ln\left[\left(\dot{\varepsilon }/D\right)\left(kT/Gb\right){\left(d/b\right)}^{2}\right]-\ln\left[\left(\sigma -\sigma_{0}\right)/G\right]$. The slope of the linear fitting line is 2.29 (≈2). The intercept of the fitting line is lnA (=6.49). Hence, A = 658.52. The determination coefficient, *R*^2^, is 0.90. Thus, the power-law constitutive equation is obtained as the following:

(8) $$\dot{\varepsilon }=658.52\times 10^{-4}\frac{Gb}{kT}{\left(\frac{b}{d}\right)}^{2}{\left(\frac{\sigma -\sigma_{0}}{G}\right)}^{2.29}exp\left(\frac{-162020}{RT}\right)$$

## 4. Discussion

### 4.1. Relationship between Microstructure and Mechanical Properties at Room Temperature

Microstructure refinement has a direct influence on mechanical properties at room temperature. As shown in Section 3.1, due to asymmetric rolling, grains in the microstructure are greatly refined compared to the as-cast microstructure. As a result, casting defects are removed due to rolling forming, and the mechanical properties of the asymmetric rolling alloy improve remarkably compared with those of the as-cast alloy. Moreover, due to imposed FSP after asymmetric rolling, because of intense, severe plastic deformation and dynamic recrystallization, dual-phase grain sizes are refined greatly. According to the well-known Hall-Petch relation, the yield strength is inversely proportional to the square root of grain size. That is to say that grain refinement increases yield strength. Hence, the mechanical properties following FSP further enhance compared to those of asymmetric rolling. 

On the other hand, various strengthening mechanisms improve yield strength in the LASY9400 alloy. In the as-cast state, the interaction between solvent Mg and solutes like Al, Sr, and Y leads to lattice distortion and increases the resistance to dislocation motion in the α-Mg and β-Li solid solution. Thus, solid solution strengthening occurs. Asymmetric rolling exerts shear strain on the alloy, increases the dislocation density, enhances the flow stress, and results in strain hardening or dislocation strengthening. Asymmetric rolling and FSP lead to pronounced grain refinement, increase the number of grain boundaries, increase the resistance of grain boundary to dislocation motion, and result in Hall-Petch strengthening or grain boundary strengthening. Finally, the second phases, such as Al_2_Y, Al_4_Sr, MgLi_2_Al, and AlLi intermetallic compounds, hinder the dislocation motion and cause Orowan strengthening during the processing process. Thus, solid solution strengthening, dislocation strengthening, grain boundary strengthening, and Orowan strengthening contribute to the yield strength and improve the load-bearing capability of the present alloy. 

Cooling modes of FSP have a certain influence on mechanical properties. Different from available reports on the pronounced effect of liquid nitrogen FSP on WE54 Mg alloy [22,44], the cooling media such as air, liquid nitrogen, and water moderately induce grain refinement in the LASY9400 alloy, probably because this alloy is a β-Li-dominated dual phase alloy in which the β-Li phase is easily dynamically recrystallized under applied FSP, and the grain size has reached the minimum grain size after FSP under present experimental conditions. Thus, the cooling medium cannot impose a remarkable influence on the grain refinement due to the softening nature in the β-Li phase during FSP. Owing to the softening nature in the β-Li phase during FSP, the nitrogen-FSP and water-FSP obtain higher ductility or elongation at room temperature. It is worth noting that the grain size treated by nitrogen-FSP is smaller than the grain size treated by water-FSP, which is smaller than the grain size treated by air-FSP. However, the mechanical properties do not vary much because of the cause mentioned above. Since the processing history has an important impact on the microstructure and mechanical properties, the FSP of multicomponent magnesium-lithium alloy in different cooling media is worth further studying in the future. 

### 4.2. Relationship between Microstructure and Ductility at Elevated Temperatures

The microstructures, such as grain size and shape, phase proportion, solutes, and grain coarsening, have a direct influence on the ductility at elevated temperatures. Firstly, the grains are equiaxed and fine below 10 μm. This fine-grained microstructure provides a prerequisite for inducing more than 400% elongation. Also, fine and equiaxed grains contain a large number of high-angle grain boundaries, which facilitate grain boundary sliding. Microscopic grain boundary sliding gives large macroscopic strain. Thus, the maximum elongation of 416% is demonstrated at 573 K and 1.67 × 10^−3^ s^−1^ in this alloy. Secondly, the phase proportion of α-Mg to β-Li in Figure 5 is 22.1:77.9, indicating that the present alloy is a β-Li matrix dual-phase alloy. This phase proportion of 22.1:77.9 deviates from the ideal phase proportion of 50:50, as demonstrated in the Mg-8Li superplastic alloy [45], where the “Crane effect” with equal phase proportion leads to the maximum superplastic elongation in a microduplex alloy. Thus, 416% ductility instead of 920% ductility [45] is obtained due to the difference in phase proportion; the former grain size is 4.12 μm and the latter grain size is 3.48 μm in the initial microstructure. Thirdly, the LASY9400 alloy is a complex system multicomponent alloy instead of a simple system alloy and is suitable for superplastic forming applications. The stress concentration caused by the dislocation pile-up at the grain boundary promotes the development of cavitation. Additionally, the interaction between dislocation motion and solute elements such as Al, Sr, and Y hinders the grain boundary sliding. As a result, the LASY9400 alloy exhibits 416% ductility in this complex system alloy rather than the 920% ductility in our previous simple system Mg-8Li alloy. That is another reason for the maximum elongation of 416%. Fourthly, as shown in Figure 6c,d, grain coarsening results in strain hardening at 573 and 623 K, and flow stress curves rise upward. Due to deformation-induced grain coarsening known during superplasticity, a certain grain growth in this alloy favors accommodation of crystal plastic equilibrium because superplasticity is a thermal activation process, and the occurrence of superplastic grain coarsening can be observed in superplastic magnesium-lithium alloys. As per Table 5, the lattice diffusion coefficient of this alloy at 573 K is 97.46 times as much as that of this alloy at 473 K, whereas the grain boundary diffusion coefficient of this alloy at 573 K is 20.75 times as much as that of this alloy at 473 K. Since the Einstein equation *B* = *D*/k*T*, where *B* is the atomic mobility, *D* is the diffusion coefficient, *k* is Boltzmann’s constant, and *T* is the absolute temperature, the migration velocity of grain coarsening is directly proportional to the atomic mobility. Thus, the grain coarsening velocity is directly proportional to the diffusion coefficient. This indicates the accelerated atomic diffusion at 573 K, which is the atomic diffusion cause of grain coarsening at 573 K in this alloy. 

As shown in Figure 6, the peculiar hardening curve at 523 K and 1.67 × 10^−4^ s^−1^ and the peculiar softening curve at 573 K and 1.67 × 10^−2^ s^−1^ are analyzed. The peculiar hardening curve at 523 K and 1.67 × 10^−4^ s^−1^ is because at a lower strain rate, dynamic grain coarsening occurs, dislocation density increases, and flow stress increases. The peculiar softening curve at 573 K and 1.67 × 10^−2^ s^−1^ is because at 573 K and higher strain rate, thermal activation accelerates, atomic diffusion enhances, and stress is relaxed. As a result, the flow stress decreases.

### 4.3. Deformation Mechanism at Elevated Temperatures

As shown in Section 3.3, the true stress exponent is 2.29 (≈2) (strain rate sensitivity 0.44), indicating the occurrence of boundary sliding. This boundary sliding is associated with (α-Mg/α-Mg, β-Li/β-Li) grain boundary sliding and (α-Mg/β-Li) interphase sliding. The experimental activation energy is 162.02 kJ/mol, larger than the theoretical activation energy of 105.77 kJ/mol determined in this alloy. Higher experimental activation energy is because the second phases, such as Al_2_Y, Al_4_Sr, MgLi_2_Al, and AlLi intermetallic compounds and grain coarsening at 573 and 623 K increase the difficulty of alloy deformation and increase the resistance to the dislocation activity as an accommodation mechanism of boundary sliding. Thus, the dominant deformation mechanism in this alloy at elevated temperatures is grain boundary and interphase boundary sliding controlled by lattice diffusion. 

## 5. Conclusions

(1) A new fine-grained LASY9400 alloy has been fabricated by asymmetric rolling and friction stir processing. Grain refinement with an average grain size in the α-Mg phase of 1.65 μm and an average grain size in the β-Li phase of 4.24 μm has been achieved in the water-cooled FSP alloy. For room-temperature performance, the ultimate tensile strength of 208 ± 4 MPa, yield strength of 193 ± 2 MPa, and elongation of 48.2% were obtained in the water-cooled FSP alloy. XRD and EDS analyses revealed that the present alloy consists of α-Mg and β-Li phases, and Al_2_Y, Al_4_Sr, MgLi_2_Al, and AlLi intermetallic compounds.
(2) For high-temperature performance, the maximum superplasticity or ductility of 416% was demonstrated in this fine-grained alloy with an average grain size of 10 μm at 573 K and 1.67 × 10^−3^ s^−1^.
(3) A power-law constitutive equation was established. The stress exponent was 2.29 (≈2) (strain rate sensitivity 0.44), and the deformation activation energy was 162.02 kJ/mol. This evidence confirmed that the dominant deformation mechanism at elevated temperatures is grain boundary and interphase boundary sliding controlled by lattice diffusion.
(4) Statement of novelty and outstanding achievements: In this report, a novel fine-grained LASY9400 alloy has been fabricated by our innovative asymmetric rolling and friction stir processing technique. Reasonable room-temperature properties and high-temperature superplasticity were obtained. The effect of cooling media on FSP microstructure and properties is rarely reported in the Mg-Li alloy system. The diffusion mechanism of grain coarsening has been quantified and elucidated. The striking achievement is to use our formulae of diffusivity and activation energy established in a simple system binary Mg-Li alloy to this complex multicomponent alloy. Another striking achievement is that the dislocation climb is revealed via a normalized plot in this multicomponent alloy at elevated temperatures. The processing, properties, and established constitutive equation can be applied to the engineering process and superplastic forming control process in the manufacturing field.

## Figures and Tables

**Table 1:** Grain refinement and superplasticity of Mg-Li based alloys.

Alloy (wt.%)	Processing	Grain Size/μm	T/K	Strain Rate/s^−1^	Elongation/%	Reference
Mg-4.07Li-3.1Al-3.4Zn-0.14Si	Extrusion	-	523, 573, 623	0.0001, 0.001, 0.01, 0.1, 1	-	[5]
Mg-8Li	ECAP *	1–7	473	1.5 × 10^−4^	1780	[6]
Mg-9Li-1Zn	HRDSR **	1–2	473, 523	2.4 × 10^−4^, 1 × 10^−3^	470~550	[7]
Mg-2.76Li-3Al-2.6Zn-0.39Y	MDF + Rolling	15.3	633	1.67 × 10^−4^	223.0	[8]
Mg-12Li-1Zn	ECAP	8	-	-	-	[9]
Mg-10.73Li-4.49Al-0.52Y	ECAP	26.4	623	5 × 10^−2^	306.6	[10]
Mg-8Li	Extrusion	-	473	10^−3^	1330	[11]
Mg-8Li	SPD	460 nm	0.35T_m_	10^−3^	440	[12]
Mg-8Li-5Zn	Extrusion + Rolling	-	473	0.001	1400	[13]
Mg-7.99Li-5.3Cd-4.57Al	Cold-rolling	33.7	423	2 mm/min	15.3	[14]
Mg-10.10Li-2.98Al-3.12Zn-0.22Si	Extrusion	10.71	-	-	-	[15]

* ECAP: Equal channel angular pressing; ** HSRDSR: High ratio differential speed rolling.

**Table 2:** Grain refinement and superplasticity of magnesium alloys.

Alloy	Processing	Grain Size/μm	*T*/K	Strain Rate/s^−1^	Elongation/%	Reference
ZK60	Submerged FSP	1.84	673	1 × 10^−2^	1205	[22]
E675	FSP	1	723	5 × 10^−2^	1300	[23]
WE54	FSP	0.9	673	1 × 10^−2^	726	[25]
AZ91	FSP	3	573	1 × 10^−4^	1604	[26]
Mg-9.4Gd-4.1Y-1.2Zn-0.4Zr	FSP	4.7	698	3 × 10^−2^	3570	[27]

**Table 3:** Room-temperature mechanical properties under different conditions in LASY9400 alloy.

Processing	UTS/MPa	YS/MPa	El/%
As-cast	141 ± 2	133 ± 4	8.2
AR	164 ± 3	113 ± 3	27.3
Air-FSP	215 ± 3	207 ± 2	25.4
Nitrogen-FSP	212 ± 2	202 ± 1	44.6
Water-FSP	208 ± 4	193 ± 2	48.2

**Table 4:** Grain sizes in gauge section of the present alloy under different micrometer conditions.

$\dot{\varepsilon }$/s^−1^				
*T*/K	1.67 × 10^−2^	1.67 × 10^−3^	5.0 × 10^−4^	1.67 × 10^−4^
473	4.07	3.98	3.86	3.81
523	4.33	4.54	3.95	6.43
573	6.81	10.05	15.62	17.35
623	14.25	17.12	34.14	39.28

**Table 5:** Theoretical activation energy and diffusion coefficients of the present alloy at different temperatures.

*T* (K)	*Q*_gb_ (kJ/mol)	*D*_gb_ (m^−2^·s^−1^)	*Q_l_* (kJ/mol)	*D_l_* (m^−2^·s^−1^)
473	68.34	2.82053 × 10^−12^	105.42	2.27978 × 10^−16^
523	68.35	1.48503 × 10^−11^	105.66	2.79971 × 10^−15^
573	68.36	5.85195 × 10^−11^	105.89	2.22186 × 10^−14^
623	68.37	1.85 × 10^−10^	106.12	1.26612 × 10^−13^

## Data Availability

The data presented in this study are available on request from the corresponding author.
